# Interference of the Histone Deacetylase Inhibits Pollen Germination and Pollen Tube Growth in *Picea wilsonii* Mast

**DOI:** 10.1371/journal.pone.0145661

**Published:** 2015-12-28

**Authors:** Yaning Cui, Yu Ling, Junhui Zhou, Xiaojuan Li

**Affiliations:** 1 Key Laboratory of Genetics and Breeding in Forest Trees and Ornamental Plants, College of Biological Sciences and Biotechnology, Beijing Forestry University, Beijing, China; 2 National Engineering Laboratory for Tree Breeding, College of Biological Sciences and Biotechnology, Beijing Forestry University, Beijing, China; Iowa State University, UNITED STATES

## Abstract

Histone deacetylase (HDAC) is a crucial component in the regulation of gene expression in various cellular processes in animal and plant cells. HDAC has been reported to play a role in embryogenesis. However, the effect of HDAC on androgamete development remains unclear, especially in gymnosperms. In this study, we used the HDAC inhibitors trichostatin A (TSA) and sodium butyrate (NaB) to examine the role of HDAC in *Picea wilsonii* pollen germination and pollen tube elongation. Measurements of the tip-focused Ca^2+^ gradient revealed that TSA and NaB influenced this gradient. Immunofluorescence showed that actin filaments were disrupted into disorganized fragments. As a result, the vesicle trafficking was disturbed, as determined by FM4-64 labeling. Moreover, the distribution of pectins and callose in cell walls was significantly altered in response to TSA and NaB. Our results suggest that HDAC affects pollen germination and polarized pollen tube growth in *Picea wilsonii* by affecting the intracellular Ca^2+^ concentration gradient, actin organization patterns, vesicle trafficking, as well as the deposition and configuration of cell wall components.

## Introduction

Pollen grains serve an important function in delivering sperm nuclei to the female gametophyte by generating pollen tubes during sexual reproduction in plants [1]. This reproductive cell is a model system in molecular and cellular studies. The tip-growing pollen tubes have a restricted expansion site associated with vesicle trafficking [2], actin cytoskeleton organization [3], apical ion flux [4], cytosolic pH, and the cytosolic Ca^2+^ gradient [5].

Chromatin is a highly complex structure of DNA and nucleo protein and can be dynamically modified during physiological processes [6]. Among these delicate modifications, acetylation and deacetylation of the lysine residues of core histones play important roles in the regulation of gene expression and chromatin state [7,8]. Histone acetylation is catalyzed by histone acetyltransferases (HATs). Targeted recruitment of HATs to a promoter is considered to be linked with histone hyperacetylation and transcriptional activation [9,10]. In contrast, the deacetylase state is maintained by histone deacetylase (HDAC), which leads to transcriptional repression [11,12].

Recent advances in this field have revealed that HDAC is both directly and indirectly involved in many biological processes, including development, proliferation, differentiation, and cell death [13]. HDAC1-knockout mice showed increased cardiac apoptosis and proliferation, leading to a phenotype of early embryonic death, as well as severe multiplication defects and general growth retardation [14,15].

In plants, however, the extent to which HDAC regulates biological functions has been of increased interest. Previous investigations showed that HDA18, a gene encoding histone deacetylase, is a key component required for the regulatory machinery of the *Arabidopsis* root epidermis [16]. In *Arabidopsis thaliana*, knockout of RPD3-related HDAC plants showed delayed flowering, suggesting that histone deacetylation plays an important role in the vegetative-to-reproductive phase transition [17]. Recently, it has been reported that inhibiting HDAC activity in cultured male gametophytes increased the transformation of embryogenesis in both *Brassica napus* and *Arabidopsis thaliana*. Further analysis suggested that this process may be correlated with high acetylation of histones H3 and H4 [18]. However, the effect of histone acetylation on the germination and polar growth of mature pollen grains has seldom been reported.

In this study, we tested the hypothesis that HDAC is involved in pollen tube growth. We treated pollen grains with two well-known inhibitors of HDAC, and then we analyzed pollen germination and tube length. Moreover, by employing dye, fluorescence labeling, and fluorescence microscope imaging technology, we observed changes in Ca^2+^ distribution, the arrangement of actin filaments, the distribution of secretory vesicles, and the cell wall components. Our findings demonstrate that interfering HDAC can affect pollen germination, pollen tube elongation, and pollen tube tip morphology, providing clues about how HDAC controls pollen tube growth.

## Materials and Methods

### Ethics statement

No specific permits were required for the described field studies. The location is not privately owned or protected in any way, and the field studies did not involve endangered or protected species.

### Plant material

Mature pollen was collected from *Picea wilsonii* trees growing in the Botanical Garden of the Institute of Botany, Chinese Academy of Science (with the permission of the Beijing Botanical Garden Institute of Botany, Chinese Academy of Sciences), on April 14, 2014. Dried pollen grains were stored at -20°C. In vitro pollen culture was performed by liquid mass culture in an Erlenmeyer flask. After 30 min of rehydration at room temperature, pollen grains were suspended in germination media containing 12% sucrose, 0.01% Ca(NO_3_)_2_, and 0.01% H_3_BO_3_ at pH 7.0 on a shaker (120 rpm) at 25°C in the dark. TSA (catalog no. T8552; Sigma-Aldrich, St. Louis, MO, USA) was dissolved in dimethyl sulfoxide (DMSO). Sodium butyrate (NaB) (catalog no. B5887; Sigma-Aldrich) was dissolved in water. Various concentrations of TSA and NaB were added to the germination media just before the culture process. In addition, pollen grains were cultured in the presence of DMSO as a control. All working concentrations of DMSO were <0.2% (v/v).

### Observation of pollen germination and pollen tube growth

Pollen grains were considered to be germinated when the tube length was greater than the diameter of the grain. The germination rate was determined by checking at least 200 pollen grains in each of three replicates. Tube length was measured by checking at least 20 pollen tubes in each of three replicates. Pollen grains were measured under an Olympus CX31 light microscope (Tokyo, Japan) and digital images were captured using a Canon 600D camera (Tokyo, Japan).

### Fluo-3/AM labeling of Ca^2+^

Pollen tubes were cultured for 24 h and then loaded with the Ca^2+^-sensitive fluorescent dye Fluo-3/AM ester (Sigma-Aldrich). Initially, pollen tubes were incubated at 4°C for 2 h in the dark in culture media containing 20 μM Fluo-3/AM ester. Pollen tubes were then rinsed three times with the corresponding media and cultured for an additional 1 h. Then, the samples were excited at 488 nm under a Leica LSM TCS SP5 microscope (Leica Microsystems GmbH, Wetzlar, Germany) and emission signals were recorded at 500–550 nm. At least twenty to thirty pollen tubes were examined in each of three replicates.

### Fluorescence labeling of actin filaments

Labeling of the actin filaments in the pollen tubes was performed with Alexa Fluor^®^ 488 Phalloidin (catalog no. A12379; Life Technologies, Carlsbad, CA, USA) according to Ou et al. [19]. The stained samples were observed under Leica LSM TCS SP5 at an excitation wavelength of 488 nm and an emission wavelength of 500–550 nm. All images were projected along the z-axis. Labeling of the actin filaments was observed by scoring at least twenty to thirty randomly chosen pollen for each of three separate.

### FM4-64 staining to analyze vesicle trafficking in the pollen tube apex

The loading of cells with FM4-64 dye was performed by direct application to the growing pollen tubes, as described previously, with minor modifications [20]. Fluorescence of samples was detected with Leica DM2500 using excitation at 510–560 nm. Serial optical sections were performed every 1 min after dye application until the fluorescence reached saturation. Images were processed using the LAS AF Lite software. For FM4-64 staining, twenty to thirty tubes were measured per replicate, with three replicates per treatment.

### Fluorescent immunolabeling of pectins in the pollen tube cell wall

Immunolabeling of pectins in pollen tube cell walls was performed according to Chen et al. [20] with slight modifications. All the samples were excited at 488 nm under Leica LSM TCS SP5 and emission signals were collected at 500–550 nm. Controls were prepared by omitting the primary antibody. For immunolabeling of pectins, twenty to thirty pollen tubes were used for data analysis in three replicates per treatment.

### Fluorescence labeling of callose

Callose in the pollen tube cell wall was labeled with decolorized aniline blue [20]. Stained samples were examined and photographed by Leica DM2500 epifluorescence (ultraviolet excitation) using excitation wavelengths of 340–380 nm. Labeling of callose was performed in triplicate, which twenty to thirty pollen tubes were used for data analysis in each of three replicates.

## Results

### TSA and NaB inhibited pollen tube growth

**Fig 1:**
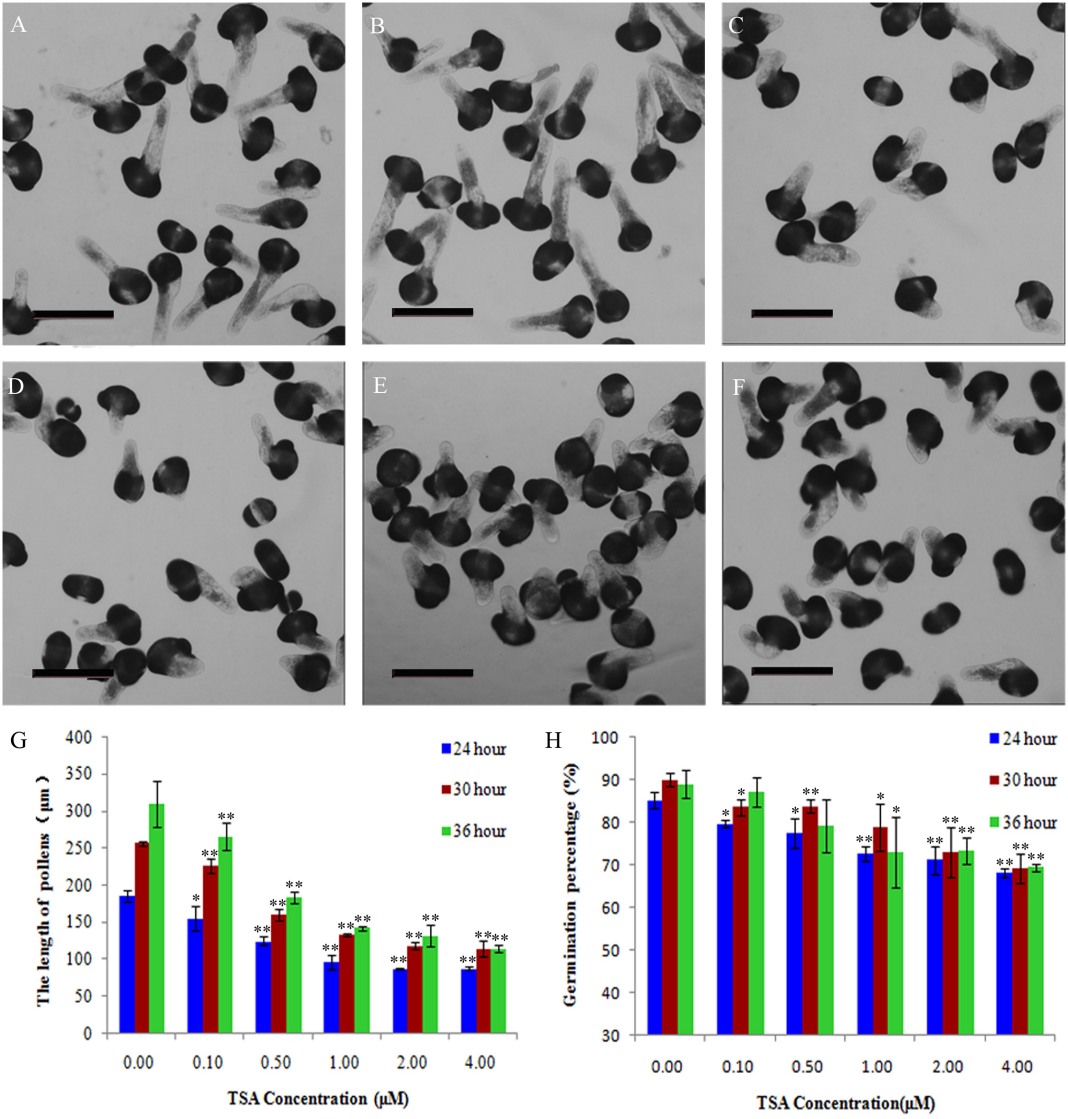
Effects of TSA on pollen germination and mean tube length in *Picea wilsonii*. Pollen germination and tube elongation were inhibited in a dose-dependent manner. Pollen tubes were cultured in (A) standard medium for 24 h, (B) medium containing 0.1 μM TSA for 24 h, (C) medium containing 0.5 μM TSA for 24 h, (D) medium containing 1 μM TSA for 24 h, (E) medium containing 2 μM TSA for 24 h, and (F) medium containing 4 μM TSA for 24 h. (G) Percent germination of pollen tubes cultured in media containing various TSA concentrations. (H) Mean lengths of pollen tubes cultured in media containing various TSA concentrations. One asterisk indicates a significant difference between the growth rates of the drug-treated and control groups at P<0.05. A double asterisk indicates a significant difference between the growth rates of the drug-treated and control groups at P<0.01. Bar = 200 μm.

As this study aimed to characterize the role of HDAC in living pollen, we first observed pollen tube growth. *Picea wilsonii* pollens were cultured in media containing various concentrations of TSA (0–4 μM). We found that, under inhibitor-free conditions, most pollen germinated and elongated after 24 h (Fig 1A). In contrast, following treatment with TSA, pollen germination and tube growth were inhibited (Fig 1B–1F). Quantitative analysis of these images showed that TSA inhibited pollen tube growth in a dose-dependent manner (Fig 1H). To further test the long-term effect of TSA, we recorded pollen germination after 24, 30, and 36 h (Fig 1G). Although the germination rates after TSA treatment were significantly lower than under the control condition, more pollen grains germinated in response to TSA over time. As expected, tube growth showed the same tendency over time, suggesting that pollen germination and tube length can be delayed by TSA treatment. It should be noted that TSA significantly inhibited pollen germination at a concentration of 0.1 μM (Fig 1G and 1H). However, it was found that 1, 2, and 4 μM of TSA had similar inhibitory effects (Fig 1G and 1H). Therefore, we can speculate that low concentrations of TSA have obvious inhibiting effects on the pollen, and the inhibition power gradually increased with the increasing concentration up to a certain maximum concentration.

**Fig 2:**
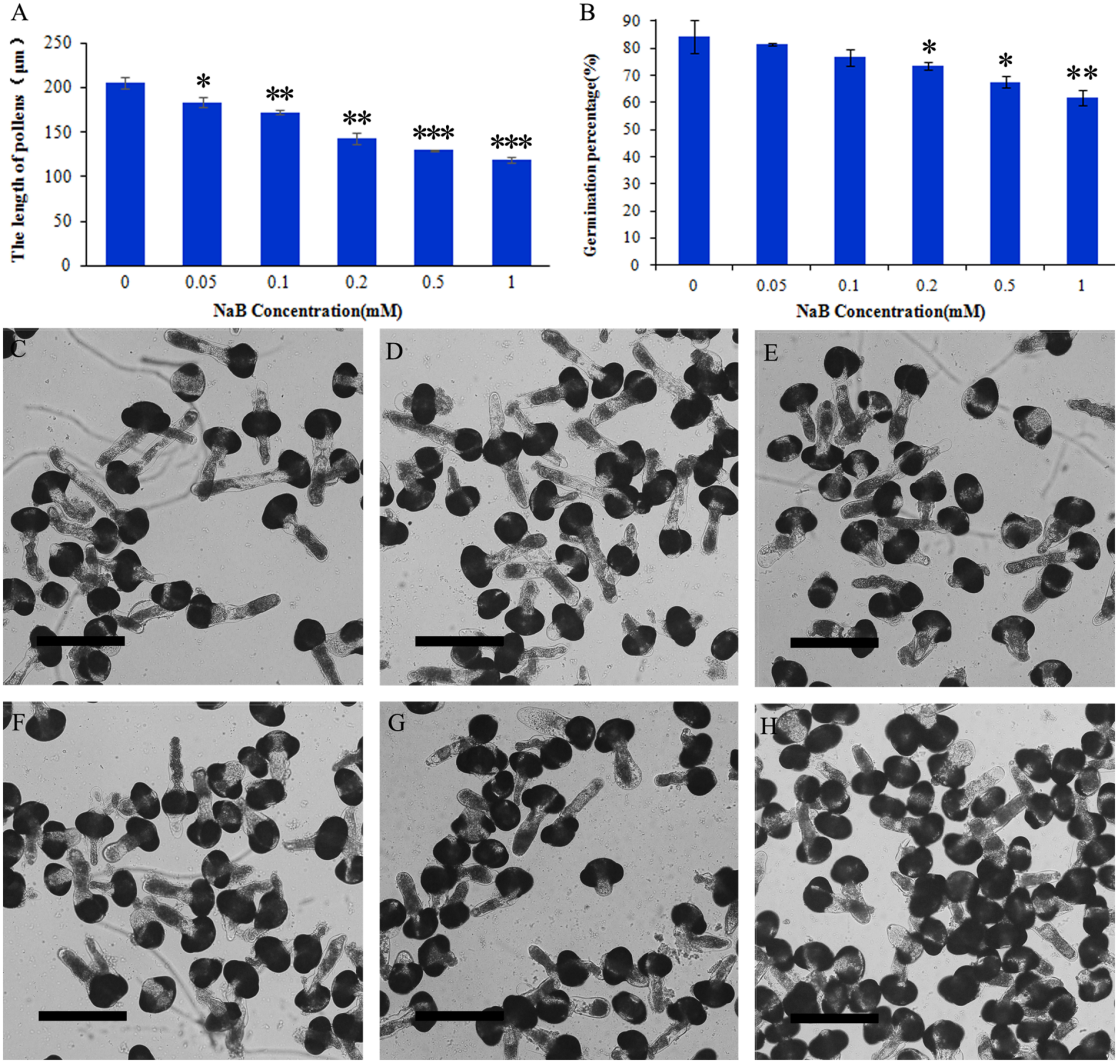
Effects of NaB on *Picea wilsonii* pollen germination and mean tube length. (A) Effects of NaB on pollen tube growth. Mean length of pollen tubes treated with 0, 0.05, 0.1, 0.2, 0.5, and 1 mM NaB, respectively. (B) Effects of NaB on pollen tube germination. Percent germination of pollen tubes treated with 0, 0.05, 0.1, 0.2, 0.5, and 1 mM NaB, respectively. (C) Pollen tubes cultured in standard medium for 24 h. (D) Pollen tubes cultured in medium containing 0.05 mM NaB for 24 h. (E) Pollen tubes cultured in medium containing 0.1 mM NaB for 24 h. (F) Pollen tubes cultured in medium containing 0.2 mM NaB for 24 h. (G) Pollen tubes cultured in medium containing 0.5 mM NaB for 24 h. (H) Pollen tubes cultured in medium containing 1 mM NaB for 24 h. One asterisk indicates a significant difference between the growth rates of the drug-treated and control groups at P<0.05. A double asterisk indicates a significant difference between the growth rates of the drug-treated and control groups at P<0.01. Three asterisks indicate a significant difference between the growth rates of the drug-treated and control groups at P<0.001. Bar = 150 μm.

We also examined the impact of NaB, another HDAC inhibitor [21]. As shown in Fig 2, pollen germination and tube growth were inhibited (Fig 2) after 24 h of treatment, similar to the effect of TSA treatment.

### TSA and NaB affected the Ca^2+^ gradient at the pollen tube

**Fig 3:**
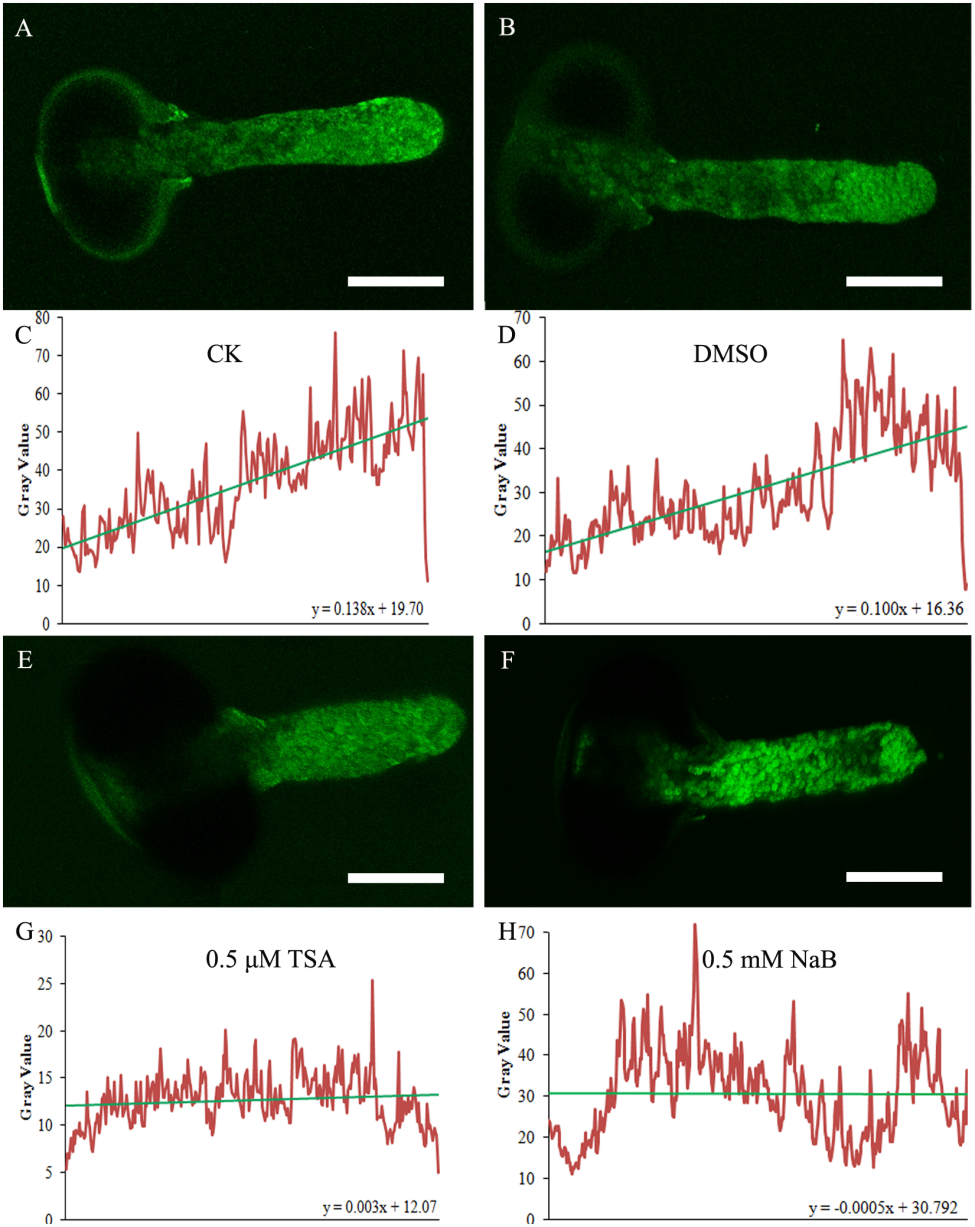
Effect of inhibitors of HDAC on cytosolic Ca^2+^ in *Picea wilsonii* pollen tubes. (A) Fluo-3/AM staining of pollen tubes cultured in standard medium for 24 h. (B) Fluo-3/AM staining of pollen tubes incubated in standard medium supplemented with 0.2% DMSO for 24 h. (C) The fluorescence intensity was analyzed using ImageJ from the base to the tip of the (A) pollen tube. The slope coefficient became larger. (D) The fluorescence intensity was analyzed using ImageJ from the base to the tip of the (B) pollen tube. The slope coefficient became larger. (E) Fluo-3/AM staining of pollen tubes incubated in medium supplemented with 0.5 μM TSA for 24 h. (F) Fluo-3/AM staining of pollen tubes incubated in medium supplemented with 0.5 mM NaB for 24 h. (G) The fluorescence intensity was analyzed using ImageJ from the base to the tip of the (E) pollen tube. The slope coefficient was largely unchanged. (H) The fluorescence intensity was analyzed using ImageJ from the base to the tip of the (F) pollen tube. The slope coefficient was largely unchanged. Bar = 50 μm.

To determine if TSA and NaB affect calcium concentration, we labeled cytosolic calcium with the calcium dye Fluo-3/AM ester at a low temperature (4°C) according to Zhang et al. [22]. Fluo-3/AM ester fluorescence in pollen tubes grown in normal culture medium was mainly located at the tip; moreover, the pollen tubes showed a typical tip-to-base cytoplasmic Ca^2+^ concentration gradient (Fig 3A). When treated with 0.2% DMSO, the pollen tubes also showed a Ca^2+^ gradient from tip to base (Fig 3B). However, the fluorescent signal gradient disappeared when pollen tubes were cultured with 0.5 μM TSA (Fig 3E). To further characterize the Ca^2+^ gradient in the tube in more detail, we obtained the fluorescence intensity from the base to the tip of the pollen tube through ImageJ analysis and then determined the slope. The greater the slope coefficient, the greater the increase in fluorescence (Fig 3C and 3D). The slope coefficient obtained under the different conditions clearly demonstrated that the Ca^2+^ gradient was completely dissipated by TSA (Fig 3G) treatment. The Ca^2+^ gradient in pollen tubes after NaB treatment was consistent with that after TSA treatment (Fig 3F and 3H).

### TSA and NaB treatment disrupted the normal arrangement of actin filaments

**Fig 4:**
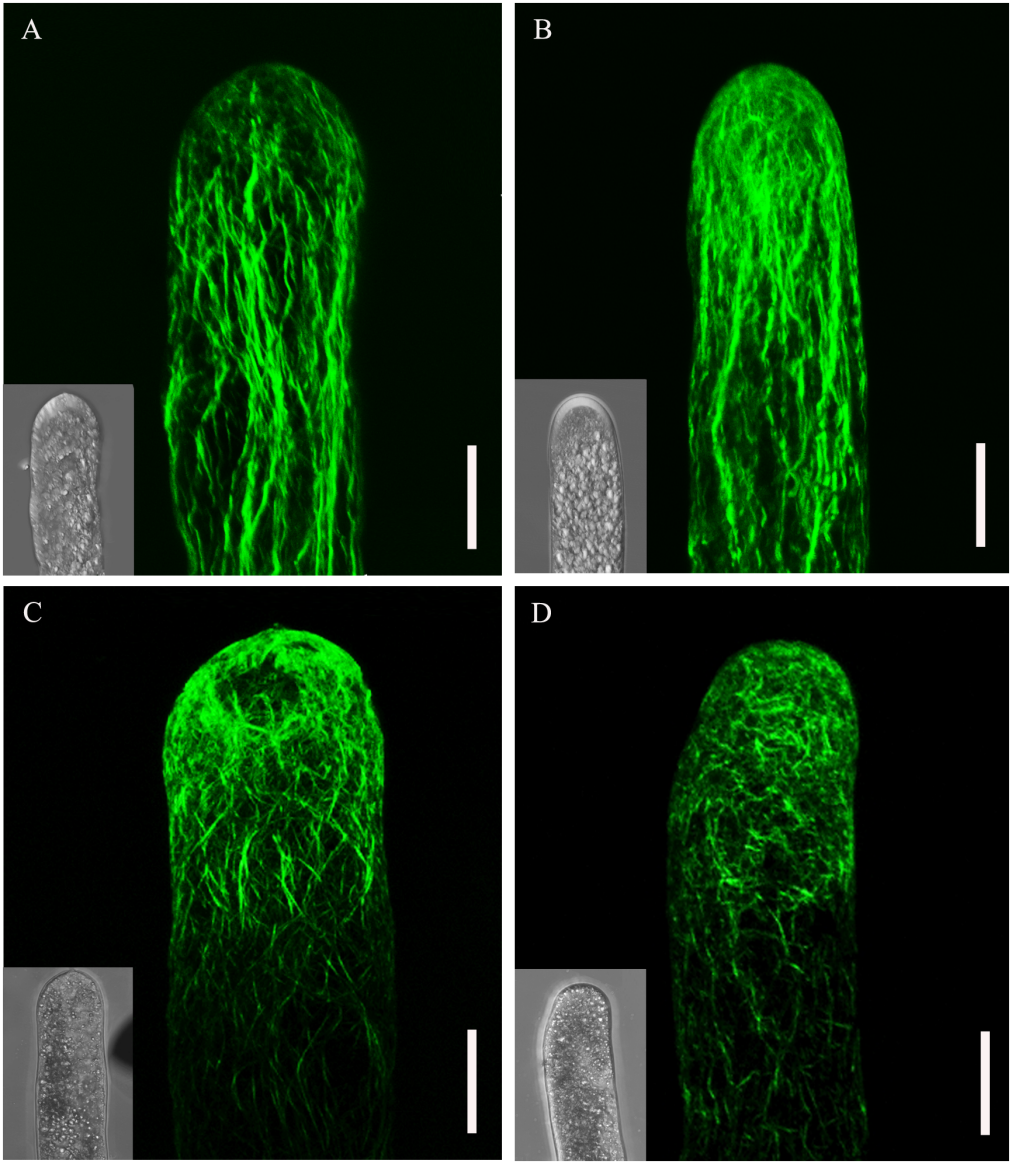
Treatment of *Picea wilsonii* pollen tubes with inhibitors of HDAC induces the redistribution of actin filaments. (A) Pollen tubes cultured in standard medium for 24 h showing actin filaments parallel to the direction of pollen tube elongation. (B) The addition of 0.2% DMSO to the standard medium followed by incubation for 24 h had no obvious effect on the distribution of the actin filaments compared with the controls. (C) In pollen tubes treated with 0.5 μM TSA-containing medium for 24 h, the actin filaments became obviously twisted and irregularly arranged. (D) In pollen tubes cultured in 0.5 mM NaB-containing medium for 24 h, the actin filaments were broken and disorganized. Bar = 20 μm.

To monitor the effect of TSA and NaB on actin filaments, pollen tubes were fixed with 4% paraformaldehyde and stained with Alexa Fluor^®^ 488 Phalloidin according to Ou et al. [19]. In control cells, we could clearly observe the actin filaments distributed in a net axial array throughout the pollen tube. The typical bundle of actin filaments was mainly parallel to the long axis, and disorganized fragments were found only in the elongating tip (Fig 4A). Furthermore, we barely detected variation in actin filament distribution in tubes cultured in DMSO at a concentration of 0.2% (Fig 4B), suggesting that DMSO did not cause artefacts. In contrast, as shown in Fig 4C and 4D, treatment with TSA and NaB caused marked twisting of the actin filaments. Moreover, fragments could be observed in the middle part of the tube, suggesting the continuous bundle initiated from the grain to the tubes was broken. These results suggest that TSA and NaB produced actin filament disorganization in pollen tubes.

### TSA and NaB disordered vesicle trafficking dynamics

**Fig 5:**
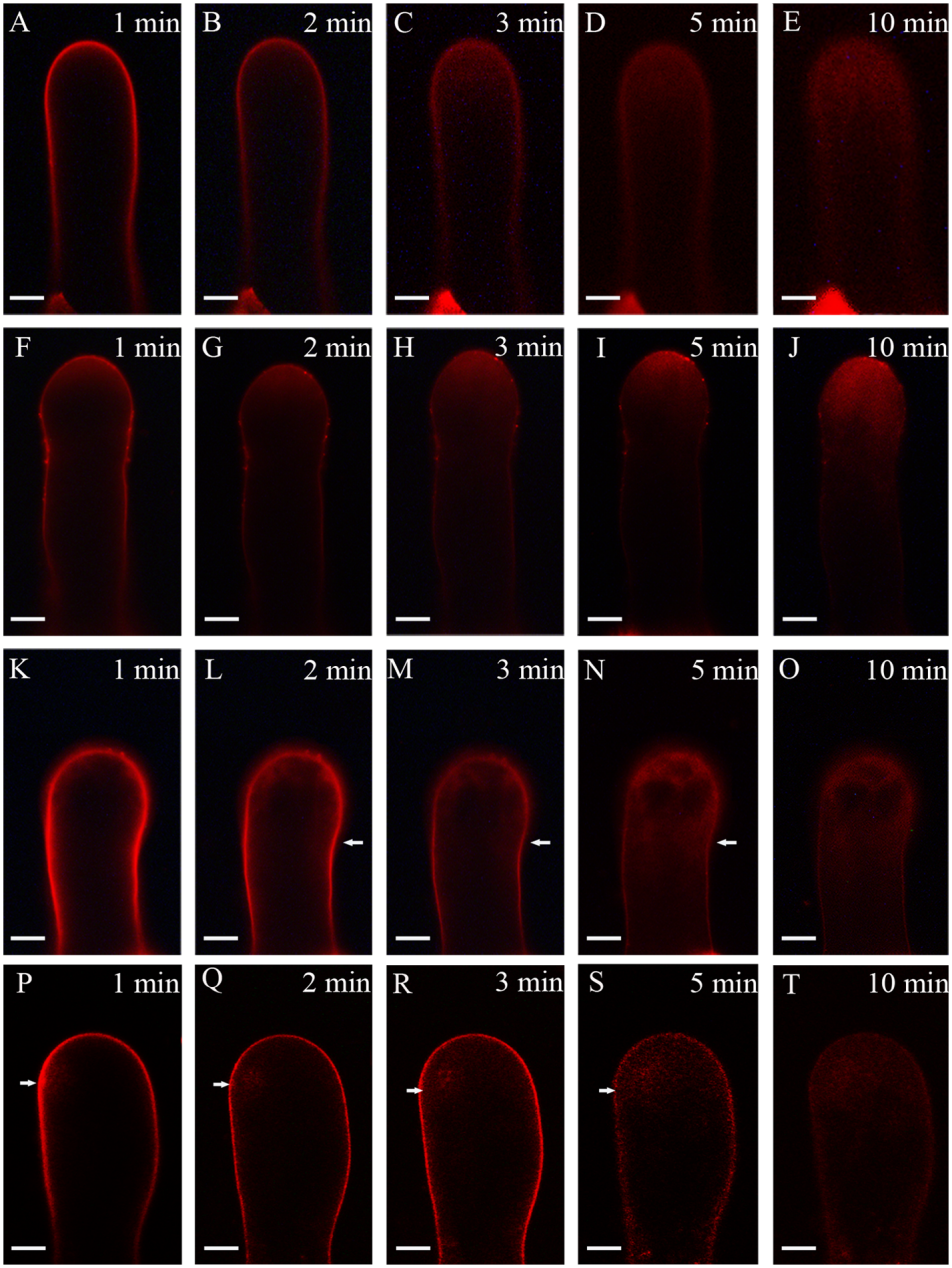
Time course of FM4-64 uptake in a growing *Picea wilsonii* pollen tube. (A–E) FM4-64 uptake into the pollen tube followed a strict time sequence, and dye uptake occurred mainly along the apex in a normally growing pollen tube of *Picea wilsonii*. (F–H) FM4-64 staining of a 0.2% DMSO-treated pollen tube. FM4-64 internalization occurred in the apical region of a growing pollen tube. (K–O) FM4-64 staining of a 0.5 μM TSA-treated pollen tube, showing nonuniform internalization from both sides of the tube. (P–T) FM4-64 staining of a pollen tube cultured in 0.5 mM NaB-containing medium for 24 h. The fluorescence was distributed unevenly and showed no distinct direction during the course of uptake. Bar = 20 μm (arrows indicate the direction of dye distribution).

Because the elongation of the pollen tube is closely related to endocytosis and exocytosis, we analyzed the vesicle trafficking under TSA and NaB treatment. Pollen tubes were stained with FM4-64, a reliable styryl dye for membrane trafficking in plant cells. As shown in Fig 5, the uptake of FM4-64 into pollen tubes was strictly time-dependent (Fig 5A–5E), resulting in a reverse V-like pattern of fluorescence in the tip of the tube after 10 min. When 0.2% DMSO was added to standard medium, pollen tubes showed similar FM4-64 internalization to those cultured in standard media (Fig 5F–5J). In contrast, when cultured in medium containing 0.5 μM TSA and 0.5 mM NaB, pollen tubes showed a different pattern of internalization. FM4-64 uptake was observed not only in the apical region, but also in the subapical region of pollen tube (Fig 5K–5T). As a result, a dispersed pattern, instead of reverse V-like pattern of dye fluorescence, was detected after TSA and NaB treatment.

### TSA and NaB treatment induced pectin distribution changes in the cell wall

**Fig 6:**
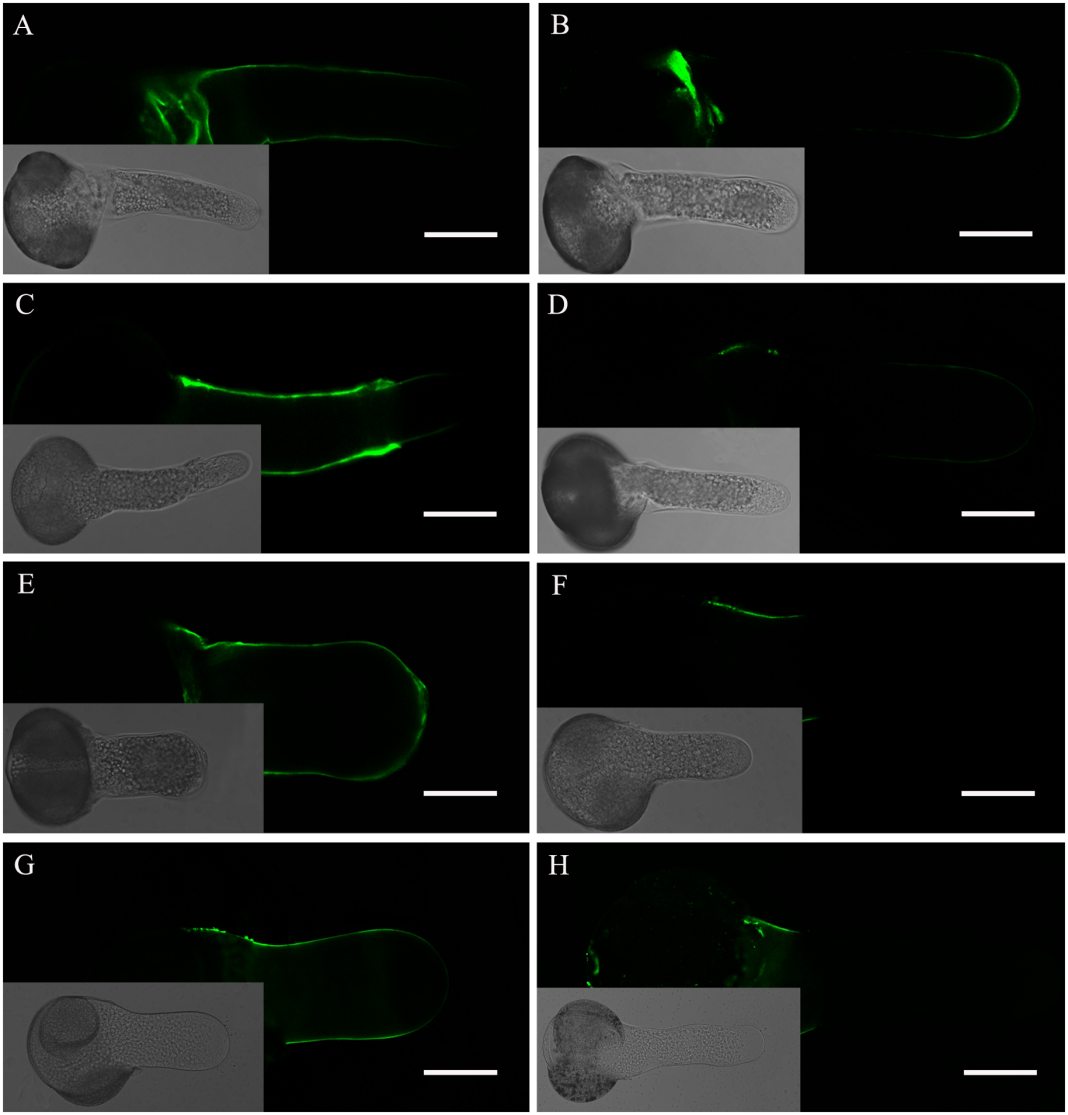
Fluorescent immunolabeling of pectins in a *Picea wilsonii* pollen tube. (A) JIM5 labeling of pollen tubes cultured in standard medium. Strong fluorescence was detected along the entire tube shank wall, excluding the tip. (B) JIM7 labeling of pollen tubes cultured in standard medium. Esterified pectins were found at the tip of the pollen tube. (C) JIM5 labeling of pollen tubes treated with medium containing 0.2% DMSO. The result is consistent with that obtained for the control (A). (D) JIM7 labeling of pollen tubes treated with medium containing 0.2% DMSO. The distribution of esterified pectins is consistent with that seen in the control (B). (E) JIM5 labeling of pollen tubes cultured in the presence of 0.5 μM TSA. The fluorescence signal was distributed along the pollen tube wall. (F) JIM7 labeling of pollen tubes incubated in medium containing 0.5 μM TSA. Esterified pectins were found only in the basal part of the pollen tube wall. (G) JIM5 labeling of pollen tubes cultured in the presence of 0.5 mM NaB. (H) JIM7 labeling of pollen tubes incubated in medium containing 0.5 mM NaB. Bar = 50 μm.

As the cell wall is a key structure in pollen tube growth, it is important to confirm whether TSA and NaB treatment would induce distribution changes of pectin in the cell wall. JIM5 and JIM7 antibodies can recognize acidic and esterified pectins, respectively, in pollen tubes. As shown in Fig 6, in the pollen tubes grown in standard media, JIM5 fluorescence was distributed along the tube shank wall, except at the apical region (Fig 6A), whereas JIM7 fluorescence was observed at the very tip of the growing tubes (Fig 6B). When cultured in medium containing 0.2% DMSO, JIM5 and JIM7 labeling of pollen tubes showed that the fluorescence signal distribution was not changed dramatically (Fig 6C and 6D). By contrast, when pollen tubes were cultured in medium containing 0.5 μM TSA and 0.5 mM NaB, the localization of pectins was completely altered. Acidic pectins were deposited along the entire surface of the pollen tubes, including the apical region (Fig 6E and 6G). Meanwhile, esterified pectins were found only in the basal site (Fig 6F and 6H), which was opposite to the results in control tubes.

### Effect of TSA and NaB on callose deposition

**Fig 7:**
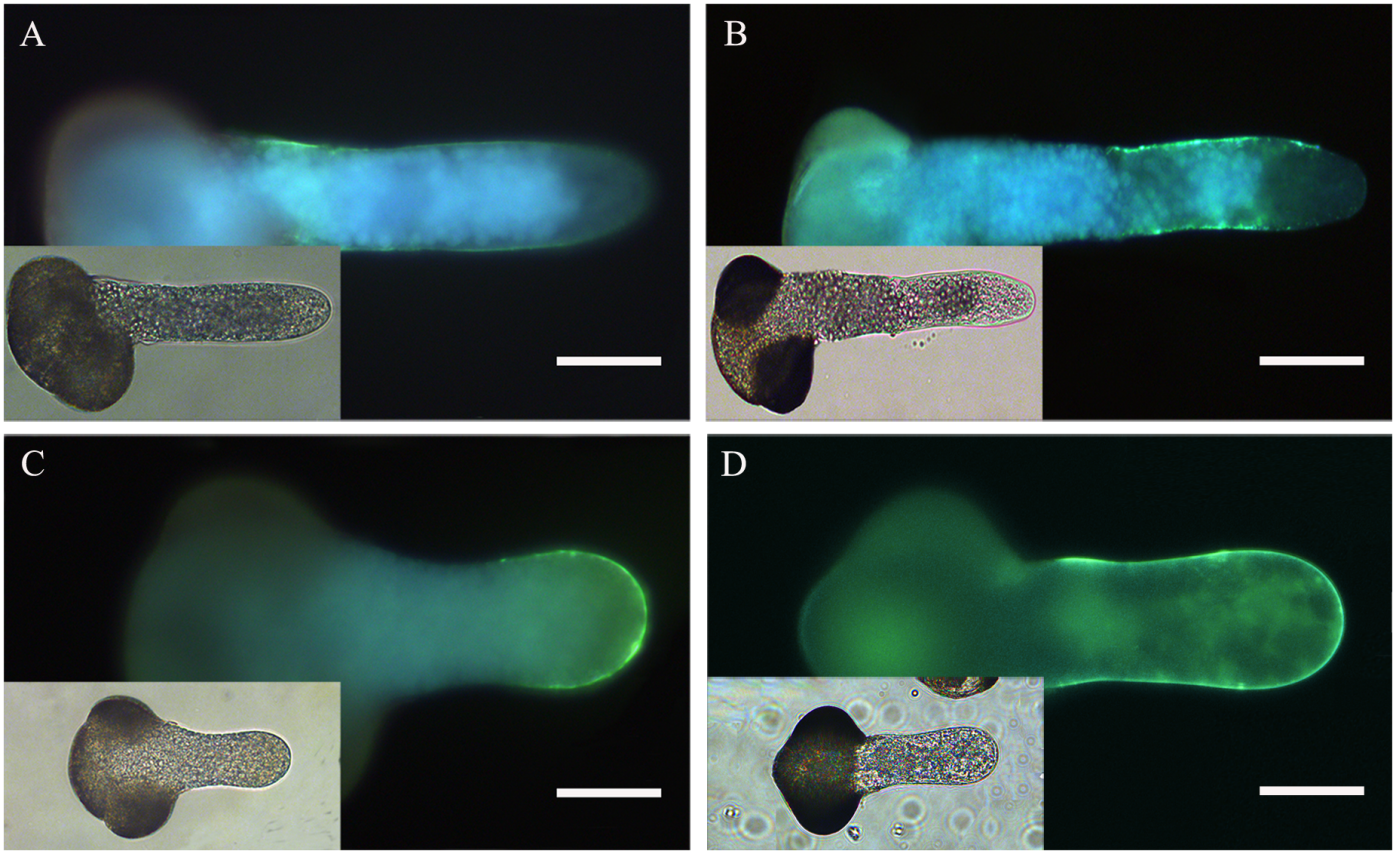
Effect of inhibitors of HDAC on callose deposition in *Picea wilsonii* pollen tube walls. (A) Control pollen tubes labeled with aniline blue. The fluorescence was distributed evenly along the tube shank, but was absent in the tip region. (B) Pollen tubes treated with 0.2% DMSO. The fluorescence distribution is consistent with that in the control. (C) Pollen tubes cultured in medium containing 0.5 μM TSA. Strong fluorescence was detected at the tip. (D) Pollen tubes cultured in medium containing 0.5 mM NaB. Bar = 50 μm.

We examined whether TSA and NaB treatment would affect callose deposition. Aniline blue produced uniform staining along the tube shank, but it was absent in the apical region in pollen tubes growing in standard media (Fig 7A). In pollen tubes cultured in media containing 0.2% DMSO, the callose fluorescence could also be found throughout the tube wall (Fig 7B), except the extreme apex, which was similar to the control. By contrast, intense fluorescence from aniline blue could be found in the apical region and subapical regions when tubes were treated with 0.5 μM TSA and 0.5 mM NaB (Fig 7C and 7D). These results show that the amount of callose in the apical region was dramatically increased by TSA and NaB treatment.

## Discussion

HDAC plays important roles in chromatin remodeling and subsequent transcriptional activation of many genes [23,24]. Previous studies have clarified the histone deacetylase was involved in pathogen response [25] and flowering [26]. In *Arabidopsis*, SWP1 represses lateral root primordium 1 (LPR1) via histone deacetylation, resulting in increased root elongation [27]. When the inhibitor of HDAC was applied to germinating *Arabidopsis* seeds, a striking increase was found in the density of root hairs of the seedlings [16]. The studies above demonstrated that histone acetylation/deacetylation status regulates many aspects of plant development. Until now, the function of the histone acetylation/deacetylation on plant reproductive process has rarely been reported. Li and colleagues proved that histone deacetylase represents a key means of embryogenesis [18], but the regulation of pollen germination and polar growth by HDAC has not been reported. In this study, we investigated the relationship between HDAC and pollen tube growth using specific inhibitors of HDAC.

Our data show that the disruption of HDAC by TSA and NaB inhibited the pollen germination ratio and tube elongation rate, suggesting that HDACs are involved in the regulation of pollen germination and tube growth. These results are consistent with an earlier study demonstrating that disruption of RNA synthesis inhibits pollen tube growth [28], further demonstrating that the dynamic changes in gene transcription activity are mediated primarily by the interwoven processes of chromatin remodeling and histone modification [29]. In addition, we found that TSA and NaB suspended pollen germination and tube growth in a concentration-dependent manner. This is identical to a previous report showing that TSA and NaB significantly inhibited primary root elongation in an approximate negative correlation to TSA and NaB concentration [21]. These results suggest that HDAC is closely linked to the cellular processes in pollen germination and pollen tube growth.

There is a close relationship between the intracellular Ca^2+^ influx and the elongation of the pollen tube. The Ca^2+^ channel is localized to a small region in the pollen tube tip, and Ca^2+^ enters from the tip and forms a gradient [30]. More recent studies have indicated that Ca^2+^ signals can increase the rate of pollen tube tip growth and facilitate a response to tropism cues [31]. Ling et al. [32] demonstrated that the Ca^2+^-dependent GABA signal can regulate pollen germination and growth. We suspect that TSA and NaB can affect the Ca^2+^ concentration gradient. To verify this hypotheses, we used the calcium dye Fluo-3/AM ester to detect cytosolic calcium. ImageJ was consistent with our assumption that TSA and NaB might regulate the change in Ca^2+^ influx. Nevertheless, there are few reports about the relationship of HDAC and Ca^2+^ in plants. Therefore, our study results suggest that calcium concentration is necessary for the HDAC-regulated polarized growth of pollen tubes.

In addition, accumulating evidence indicates that actin filaments can regulate pollen tube growth [33]. They act as a complex signaling network not only to regulate pollen tube tip growth but also for polarized diffuse growth in plants [34]. In most cases, actin dynamics can be controlled by Ca^2+^ and Ca^2+^-dependent enzymes [35]. HDAC is a key regulator of cytoskeleton components in mammalian cells [36]. It has been reported that HDAC8 associates with and regulates smooth muscle cell cytoskeleton dynamics [37]. HDAC6-mediated deacetylase activity plays a key role in the antigen-specific reorientation of the tubulin cytoskeleton [38]. Our results show that the treatment of pollen grains with TSA and NaB induce the polymerization of actin to become disorganized in the pollen tube tip region, suggesting sensitivity to TSA and NaB. Therefore, we speculate that HDAC signaling affects intracellular Ca^2+^ levels, blocking the polymerization of actin filaments in pollen tube tips and affecting tube germination.

Vesicle trafficking is fundamental to most activities in organisms, and FM4-64 staining shows a new tracer of membrane trafficking in living cells [39]. We also can assume that FM4-64 is present in endocytic vesicles [40]. Endocytosis is now considered to be a requirement for pollen tube tip growth. The results of time-course images of TSA- and NaB-treated pollen tubes showed a FM4-64 staining pattern different from that of controls. In *Picea wilsonii* pollen tubes, FM4-64 internalization occurred in the apical region, and it ultimately appeared in an inverted cone-shaped pattern, which was quite different from reports in angiosperm species [40]. In our control experiments, *Picea wilsonii* Mast. pollen tubes also formed similar inverted cone-shaped patterns in the apical region. However, in TSA- and NaB-treated pollen tubes, FM4-64 internalization occurred primarily in other sites of pollen tubes rather than in the apical region, indicating that vesicular trafficking was perturbed by the chaotic status of actin filaments. We think that HDAC may be involved in regulating vesicular trafficking in pollen tubes by modulating the actin filament pattern.

Cell wall extensibility and rigidity are key parameters in the development and architecture of plant cells [41]. In the growing pollen tube, apex pectins and callose are the primary cell wall components. Pectins in the pollen tube apex are secreted via the Golgi apparatus-based vesicles as esterified pectins, which are subsequently made acidic by pectin methylesterase in the tube wall via the endosomal recycling pathway [32]. Our studies show that, in control tubes, pectins accumulated in the tip of the growing pollen tube and were highly esterified, whereas they were de-esterified in cell walls along the length of the tubes. When compared with the controls, acidic pectins were distributed along the pollen tube wall, whereas esterified pectins reduced dramatically in the tip region and were only found in the basal part of the pollen tube after incubation with TSA and NaB. Furthermore, callose is also a small portion of the cell wall polymers, and callose deposition can be enhanced by a variety of stimuli [42]. Our data from an aniline blue staining experiment showed that callose deposition changed after TSA and NaB incubation, suggesting that HDAC affects the deposition distribution of cell wall components in growing pollen tubes. Previous studies in *Arabidopsis* also reported that a number of specific pathways were altered by TSA treatment, including genes involved in cell wall loosening/degradation, pectin depolymerization/solubilization, as well as cellulose hydrolysis[18].

In summary, our study shows that the inhibition of HDAC activity in pollen tubes promoted intracellular Ca^2+^ concentration gradient changes and altered actin filament networks. Consequently, the disorganization of vesicle trafficking and changes in cell wall components led to altered tip growth. Our analyses of the effect of TSA and NaB on gymnosperm pollen tubes provide useful information about the role of HDAC in the polarized tip growth of pollen tubes.
